# Medical students as human subjects in educational research – the importance of responder bias

**DOI:** 10.3402/meo.v18i0.20757

**Published:** 2013-05-27

**Authors:** Kieran Walsh

**Affiliations:** BMJ Group, BMA house, Tavistock Square, London WC1H 9JR, UK

Sarpel and her colleagues should be praised for bringing much-needed evidence to the debate about the degree of rigor required when conducting research on medical students (1). Their study explored ‘the perceived level of risk of studies where medical students serve as human subjects’ (1). They evaluated the response of review boards to the same study protocol and the feelings of students about being research subjects.

However, a potentially important limitation to their study is the attrition rate – which the authors mention but do not weigh sufficiently in terms of possible implications. That is, the authors do not specify whether the students in the focus groups were only those who completed the study or also included subjects who began the study but later dropped out. If, for example, the focus groups were composed of a mixture of students, then it is difficult to know whether those who dropped out fully experienced the study and, hence, were able to give a truly informed opinion.

Conversely, if students who withdrew from the study were not a random subsample, then including in the focus groups only those who completed the study may have resulted in a ‘response bias’ of sorts. This dilemma is inherent in many research studies, the weakness, if there was one, was in not explicitly and sufficiently addressing the issue.

Since the factors underlying a potential bias are numerous, and vary in their relevance to the research question, empirically discerning their impact on the validity of the study findings is typically not possible. For instance, it may be that those who dropped out simply had no desire to further participate in the study. Or, it may be that subjects who chose to withdraw were concerned with confidentiality issues, and/or that their performance might factor into a formal, summative assessment. Finally, some may have felt that they were members of a vulnerable population. For example, they may have felt stressed or worried, or they may have had educational challenges during their time at medical school. This may have biased the subsequent study findings. To give an explicit example, students concerned about confidentiality might have been more likely to withdraw; these same students may not be representative of the student body as a whole – they may have been borderline pass–fail students who did not want to risk sharing their study habits with potential assessors.

To this end, a brief description of the pre- and post-intervention participants – and their representation within the focus groups – would have been helpful in better assessing any potential threats to validity.

## References

[1] Sarpel U, Hopkins MA, More F, Yavner S, Pusic M, Nick MW (2013). Medical students as human subjects in educational research. Med Educ Online.

